# Walking economy at simulated high altitude in human healthy young male lowlanders

**DOI:** 10.1242/bio.019810

**Published:** 2016-10-15

**Authors:** Masahiro Horiuchi, Yoko Handa, Daijiro Abe, Yoshiyuki Fukuoka

**Affiliations:** 1 Division of Human Environmental Science, Mt. Fuji Research Institute, Kami-yoshida 5597-1, Fuji-yoshida-city, Yamanashi 4030005, Japan; 2 Center for Health and Sports Science, Kyushu Sangyo University, Matsukadai 2-3-1, Higashi-ku, Fukuoka-city, Fukuoka 8138503, Japan; 3 Faculty of Health and Sports Science, Doshisha University, Tatara 1-3, Kyotanabe, Kyoto 6100394, Japan

**Keywords:** Bipedal locomotion, Optimal speed, Muscle O_2_ extraction, Peripheral circulation, Energy expenditure

## Abstract

We measured oxygen consumption during walking per unit distance (*C_w_)* values for 12 human healthy young males at six speeds from 0.667 to 1.639 m s^−1^ (four min per stage) on a level gradient under normobaric normoxia, moderate hypoxia (15% O_2_), and severe hypoxia (11% O_2_). Muscle deoxygenation (HHb) was measured at the vastus lateralis muscle using near-infrared spectroscopy. Economical speed which can minimize the *C_w_* in each individual was calculated from a U-shaped relationship. We found a significantly slower economical speed (ES) under severe hypoxia [1.237 (0.056) m s^−1^; mean (s.d.)] compared to normoxia [1.334 (0.070) m s^−1^] and moderate hypoxia [1.314 (0.070) m s^−1^, *P*<0.05 respectively] with no differences between normoxia and moderate hypoxia (*P*>0.05). HHb gradually increased with increasing speed under severe hypoxia, while it did not increase under normoxia and moderate hypoxia. Changes in HHb between standing baseline and the final minute at faster gait speeds were significantly related to individual ES (r=0.393 at 1.250 m s^−1^, r=0.376 at 1.444 m s^−1^, and r=0.409 at 1.639 m s^−1^, *P*<0.05, respectively). These results suggested that acute severe hypoxia slowed ES by ∼8%, but moderate hypoxia left ES unchanged.

## Abstract (precis)

**Summary:** Acute severe hypoxia slowed the economical speed (ES) which can minimize energy cost of walking. Muscle O_2_ extraction may be one of the determining factors of an individual's ES.

## INTRODUCTION

As humans moved into high-altitude regions over the past 20,000 years, these populations adapted culturally and physiologically to the reduced availability of oxygen in the atmosphere (Beall, 2007). Cardiovascular adaptation to altitude has been primarily studied at rest (Beall, 2007), and comparatively little is known about metabolic responses during walking under hypoxia. Energy costs in Himalayan porters and Tibetan migrants were significantly lower during walking compared to lowlanders (Marconi et al., 2005; Minetti et al., 2006). Similarly, East African women (Maloiy et al., 1986) and Himalayan porters (Bastien et al., 2005) who live at high altitudes can walk and carry heavy baggage as part of their daily lives with unchanged energy cost. Although the underlying mechanisms of the lower and effective energy cost during walking in these populations remain unclear, these results suggest that chronic exposure-induced hypoxia due to sustained and specific training at high altitudes may cause specialized adaptation in these ethnic groups (Marconi et al., 2005; Minetti et al., 2006). It has also been reported that the arterial oxygen content of Tibetans is markedly lower than that of Andeans, whereas exhaled nitric oxide (NO) concentration, which is a potent vasodilator, is higher compared to Andeans and lowlanders (Beall, 2007). Additionally, native highlanders were found to have higher forearm blood flow and circulating concentrations of bioactive NO products than low-altitude residents (Erzurum et al., 2007). These results may imply that adaptation to high altitude (e.g. oxygen delivery to tissues) may be affected by vasodilator function linked to enhanced blood flow, although this interesting hypothesis has never been applied to lowlanders.

It is well known that there is a U-shaped relationship between oxygen consumption during walking per unit distance (*C_w_*; ml kg^−1^ m^−1^) and gait speeds (*v*; m s^−1^) (Saibene and Minetti, 2003). This indicates that every individual has a particular gait speed that minimizes *C_w_*, which is called the economical speed (ES) (Abe et al., 2008a,b; Horiuchi et al., 2014c, 2015a; Saibene, 1990; Wezenberg et al., 2013); however, to the best of our knowledge, no study has been conducted on the ES of individuals at simulated high-altitude.

At high altitude, it is known that peak aerobic capacity is reduced with a reduction of inspired oxygen pressure (Calbet et al., 2009). Under these conditions, it is possible that ES would be slow, because diminished aerobic capacity causes relatively higher exercise intensity. Traditionally, an individual's ES may be estimated by physical characteristics (Donelan and Kram, 1997), including height (Abe et al., 2008b) and leg length (Horiuchi et al., 2015a). Additionally, preferred walking speed, which was found to be almost consistent with ES in previous studies (Malatesta et al., 2003; Wezenberg et al., 2013), has been suggested to be related to peak aerobic capacity and muscle mitochondrial capacity (Coen et al., 2013).

There are, however, technical limitations to continuously measuring muscle mitochondrial capacity in active skeletal muscles during walking. According to the Fick equation (McArdle et al., 1996), oxygen uptake can be determined by a function of O_2_ delivery and O_2_ extraction. O_2_ extraction occurs at active skeletal muscle, which is defined as an arterial-venous O_2_ difference (*a-v* O_2_ difference). As muscle O_2_ extraction and skeletal muscle mitochondrial capacity have been related to exercise performance (Jacobs et al., 2013), the continuous measurement of muscle O_2_ extraction can provide new information toward a better understanding of the factors that explain individual ES. An alternative approach would be to measure muscle deoxygenation (HHb) derived from near infrared spectroscopy (NIRS), because changes in HHb have been considered a surrogate of microvascular O_2_ extraction (DeLorey et al., 2003; Grassi et al., 2003).

In the present study, we sought to investigate the potential impact of hypoxic conditions on an individual's ES during walking. We hypothesized that ES would be slower with a decrease in fractional inspiratory oxygen concentration (FiO_2_), and that possible alterations in ES would account for changes in muscle O_2_ extraction. To test this hypothesis, in addition to oxygen consumption measurement, muscle deoxygenation profiles (the balance between V̇O_2_ and *Q̇* in exercising muscle) at the vastus lateralis muscle were simultaneously measured by NIRS.

## RESULTS

**Fig. 1.**
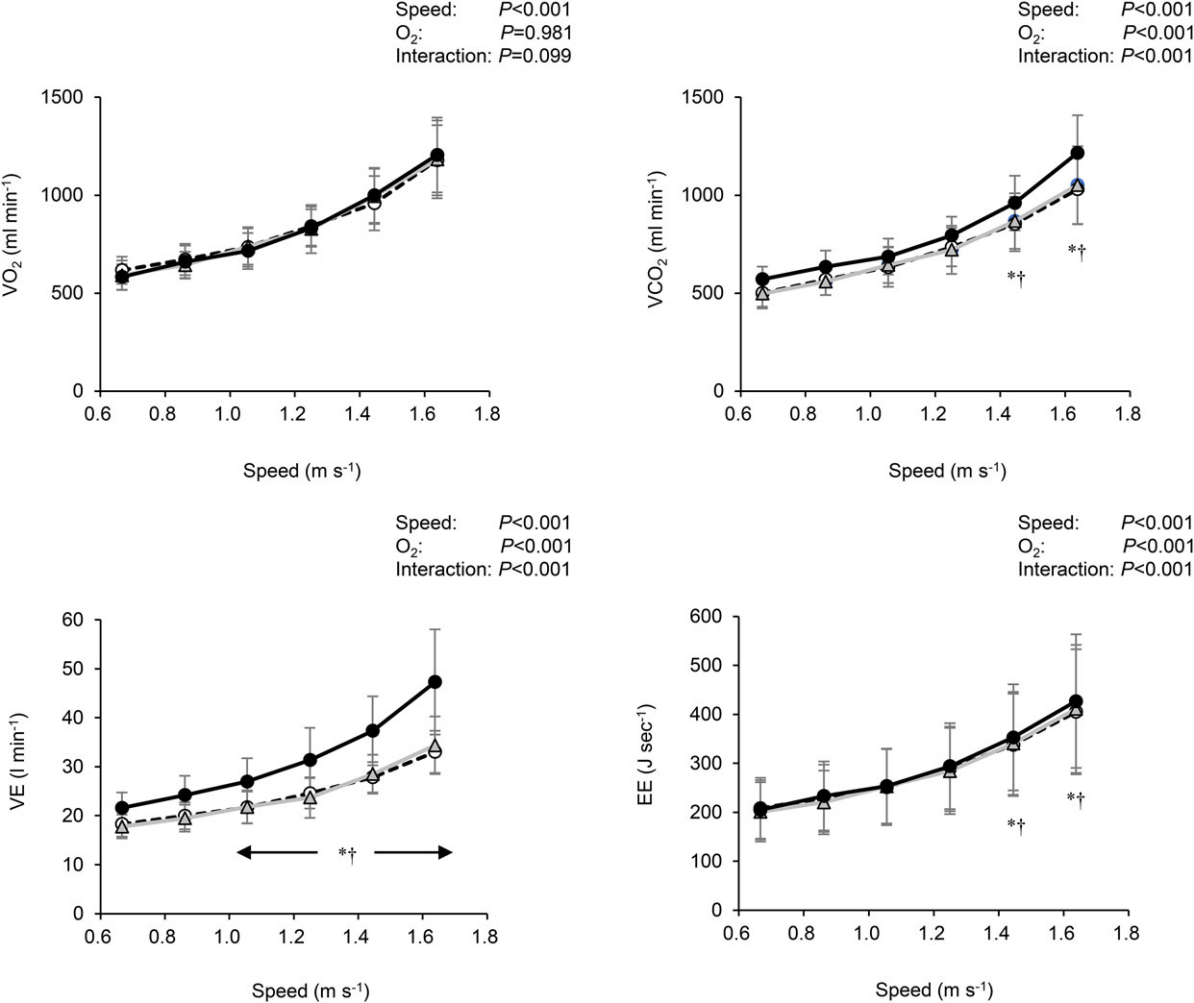
**Changes in cardiorespiratory variables and energy expenditure (EE) at all gait speeds under normoxia (○), moderate hypoxia (△), and severe hypoxia (●).** Values are mean±s.d. *P<0.05 between normoxia and severe hypoxia, †*P*<0.05 between moderate and severe hypoxia within a same speed, respectively (Tukey *post hoc* test was used). V̇O_2_, oxygen uptake; V̇CO_2_, carbon dioxide output; V̇_E_, pulmonary ventilation. Error bars indicate s.d.

Table 1 shows cardiorespiratory variables at resting baseline with different inspired-oxygen concentrations. There were no differences in pulmonary oxygen uptake (V̇O_2_), and energy expenditure (EE) between the different oxygen concentrations. Meanwhile, carbon dioxide output (V̇CO_2_), pulmonary ventilation (V̇_E_), respiratory gas exchange ratio (RER) and heart rate (HR) linearly increased as concentration of FiO_2_ fell (*P*<0.001). These parameters under severe hypoxia were significantly higher compared to normoxia and moderate hypoxia (*P*<0.05, respectively). In contrast, arterial O_2_ saturation (SpO_2_) linearly decreased with decreasing inspired O_2_ concentrations (*P*<0.001), and the differences between conditions were statistically significant (*P*<0.05). During walking, no differences in V̇O_2_ were observed among conditions at any gait speed, whereas V̇CO_2_, V̇_E_, and energy expenditure (EE) at faster gait speeds under severe hypoxia were significantly greater than normoxia and moderate hypoxia (*P*<0.05, respectively, Fig. 1).

**Table 1.**
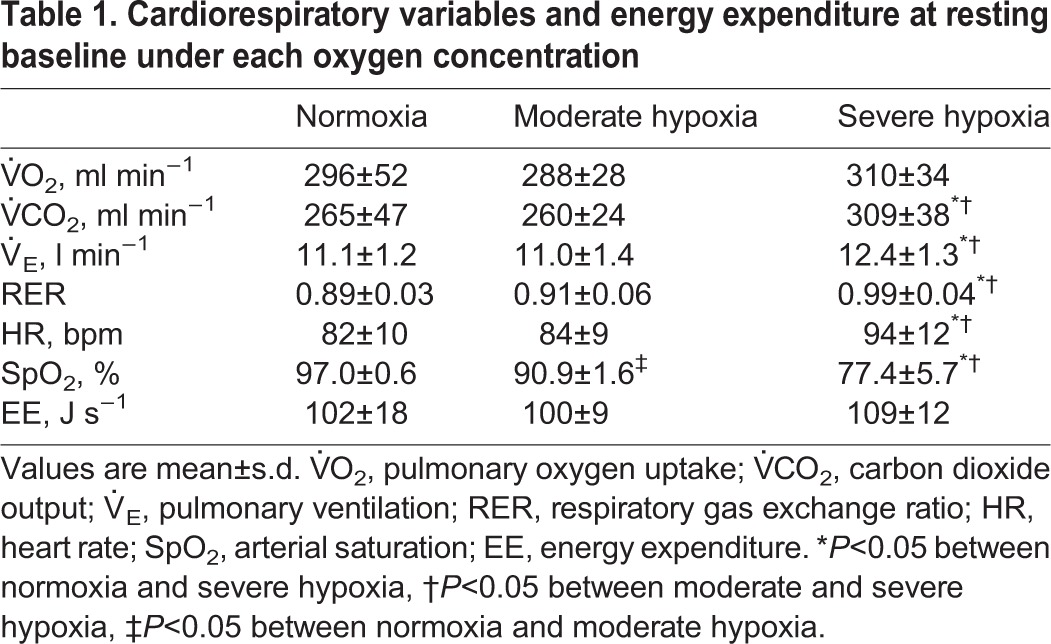
**Cardiorespiratory variables and energy expenditure at resting baseline under each oxygen concentration**

**Fig. 2.**
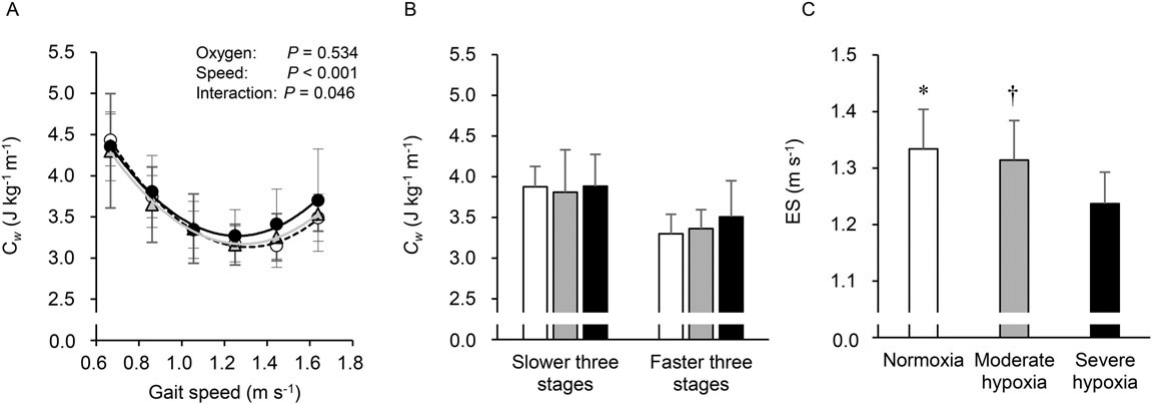
**Energy cost of walking and economical speed**. Energy cost of walking (*C_w_*) at all gait speeds (A); averaged *C_w_* during slower and faster three stages at each oxygen concentration (B); and comparisons in the economical speed (ES) at each oxygen concentration (C). White circles and bars indicate normoxia, gray triangles and bars indicate moderate hypoxia, and black circles and bars indicate severe hypoxia. In A, values are mean±s.d. Error bars indicate s.d. *P<0.05 between normoxia and moderate hypoxia, ^†^*P*<0.05 between moderate and severe hypoxia (Tukey *post hoc* test was used).

Mean values of the *C_w_* at each oxygen concentration are shown in Fig. 2A. The figure shows that the averaged correlation coefficient values of the *C_w_-v* relationship in the 36 trials of the present study (12 participants×three oxygen levels) were 0.982 (0.937∼0.999). Two-way repeated measures analysis of variance (ANOVA) revealed no significant main effect of oxygen concentrations (*P*>0.05), while significant main effects were observed for gait speed and interaction (*P*<0.05, Fig. 2A). Fig. 2B shows the averaged *C_w_* during the slower three gait speeds (between 0.667 and 1.056 m s^−1^), and faster three gait speeds (between 1.250 and 1.639 m s^−1^). From ANOVA with linear trend analysis, the averaged *C_w_* at faster three gait speeds linearly increased (*P*<0.05) among three FiO_2_ with no statistical differences in pairwise comparisons. The averaged *C_w_* at the faster gait speeds under severe hypoxia (3.51±0.44 J kg^−1^ m^−1^) was ∼6.4% greater than normoxia (3.30±0.24 J kg^−1^ m^−^^1^); meanwhile, almost equivalent values were found for *C_w_* during the slower gait speeds in all conditions (*P*>0.05). Fig. 2C illustrates the comparison of ES under the three oxygen concentrations. ES linearly decreased as concentration of FiO_2_ fell (*P*<0.001). Moreover, ES under severe hypoxia was significantly slower than under normoxia and moderate hypoxia (*P*<0.05, respectively), while no differences were observed in ES between normoxia and moderate hypoxia (1.334±0.070 m s^−1^ in normoxia, 1.314±0.070 m s^−1^ in moderate hypoxia, and 1.237±0.056 m s^−1^ in severe hypoxia). Yet there were no statistically significant differences in the coefficients *a* and *b* of the quadratic equation among conditions (*P*>0.05, Table 2).

**Table 2.**
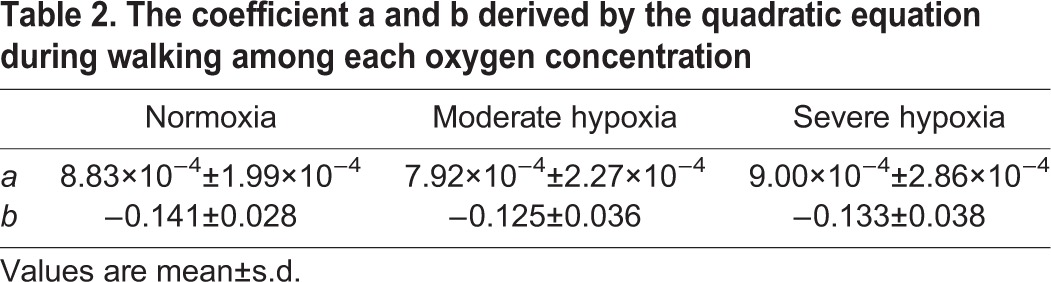
**The coefficient a and b derived by the quadratic equation during walking among each oxygen concentration**

**Fig. 3.**
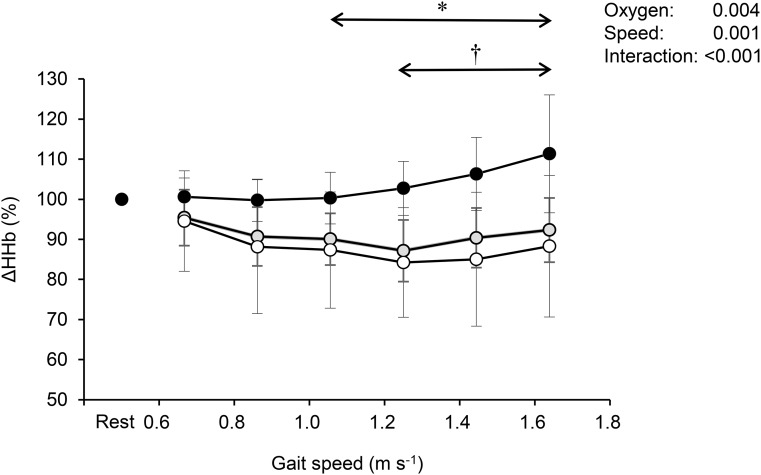
**Relative changes from standing baseline values in deoxygenated hemoglobin (HHb) corresponding to gait speed at each oxygen concentration.** White circles indicate normoxia, gray circles indicate moderate hypoxia, and black circles indicate severe hypoxia. Values are mean±s.d. *P<0.05 between normoxia and moderate hypoxia; †*P*<0.05 between moderate and severe hypoxia; (Tukey *post hoc* test was used).

Fig. 3 shows the relative changes from the standing baseline values in HHb at each gait speed. Although HHb remained unchanged at the slower speeds, it gradually increased in accordance with increasing speed under severe hypoxia; however, it stabilized under normoxia and moderate hypoxia during walking. There were significant differences in relative changes of HHb between severe hypoxia and normoxia above 1.056 m s^−1^ of gait speed (*P*<0.05). Additionally, significant differences in HHb were observed between moderate and severe hypoxia above 1.250 m s^−1^ of gait speed (*P*<0.05).

**Fig. 4.**
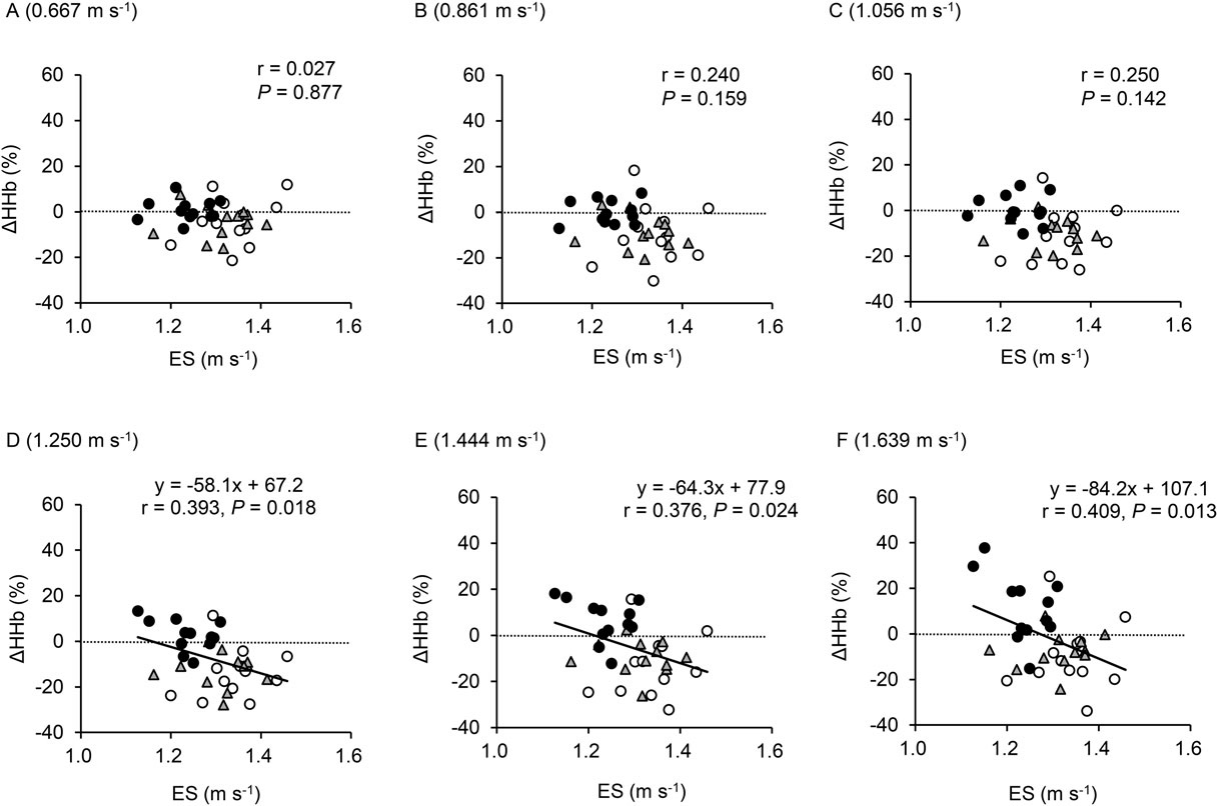
**Relationships between an individual's ES and delta changes in HHb between standing baseline and last 1-min data at each gait speed.** Data are pooled in all oxygen concentrations (*n*=36). Data points indicate normoxia (○), moderate hypoxia (△), and severe hypoxia (●).Gait speed of (A) 0.667 m s^−1^; (B) 0.861 m s^−1^; (C) 1.056 m s^−1^; (D) 1.250 m s^−1^; (E) 1.444 m s^−1^; and (F) 1.639 m s^−1^.

Fig. 4 shows the relationships between ES at each oxygen level, and changes in HHb signals from the baseline values to the last 1 min at each gait speed. Increases in HHb were significantly related to ES for the three faster gait speeds (i.e. above 1.250 m s^−1^) when the data were pooled.

## DISCUSSION

To the best of our knowledge, this is the first study to examine whether hypoxic conditions alter *C_w_* and ES during walking. The major findings of the present study were threefold. First, averaged *C_w_* at faster gait speeds linearly increased as concentration of FiO_2_ fell, although overall *C_w_* under moderate and severe hypoxia was not significantly different from normoxia. Second, ES linearly decreased as concentration of FiO_2_ fell and a significantly slower ES was observed only under severe hypoxia; there were no differences in ES between normoxia and moderate hypoxia. Finally, relative changes in HHb at the vastus lateralis muscle from standing to faster gait speeds were inversely correlated with decelerated ES. Collectively, these results suggest that the U-shaped relationship between gait speeds and *C_w_* showed only a leftward shift under severe hypoxia, resulting in a slower ES. In addition, these changes in ES might be partly accountable for local muscle O_2_ extraction.

Although no studies have investigated alterations of *C_w_* and ES under hypoxia, hypoxia by itself may reflect relatively higher exercise intensity. However, it is supposed that mechanical energy demands of walking are the same regardless of oxygen concentration, although we did not measure it. Thus, unchanged overall *C_w_*, even under severe hypoxia, may not be a surprising finding. Previous studies reported that V̇O_2_ kinetics slowed at the onset of moderate exercise while breathing hypoxic gas mixture (12∼15% O_2_); these studies, however, also demonstrated that V̇O_2_ showed a stable phase 1∼2 min after the onset of exercise with no differences between normoxia and moderate hypoxia (DeLorey et al., 2004; Engelen et al., 1996). These results suggested that hypoxia by itself did not affect steady-state whole-body V̇O_2_, resulting in unchanged *C_w_* values (Fig. 2A).

Nevertheless, it should be noted that effects of hyperventilation-induced higher V̇CO_2_ under severe hypoxia caused higher EE at faster speeds under severe hypoxia than normoxia and moderate hypoxia (Fig. 1). Therefore, it is still possible that *C_w_* may be influenced by higher V̇CO_2_. We found significant linear increases in averaged *C_w_* during the faster gait speeds (1.250∼1.639 m s^−1^) as concentration of FiO_2_ fell. Specifically, the averaged *C_w_* above 1.250 m s^−1^ under severe hypoxia was 6.4% higher than normoxia despite no significant differences in pairwise comparisons (Fig. 2B). This higher *C_w_* during faster walking would lead to a steeper quadratic curve, resulting in a higher coefficient *a*. It was notable that the difference in the averaged ES between normoxia and severe hypoxia was about 7.8% (1.334 vs 1.237 m s^−1^), indicating that a considerable 6.4% higher *C_w_* during the faster gait speeds under severe hypoxia could explain the different ES. Otherwise, as the ES was determined by the coefficients *a* and *b* [see Eqn (5) in the method section], greater coefficient *a* and/or lesser coefficient *b* resulted in slower ES (Abe et al., 2015). We found no significant differences in the coefficients *a* and *b* among conditions, because the coefficients *a* and *b* are determined from *C_w_* values observed at six gait speeds. Indeed, the coefficient *a* under severe hypoxia was higher than normoxia by ∼2%, and the coefficient *b* under severe hypoxia was lower than normoxia by ∼6% (Table 2). Taken together, the fact that the slower ES was unchanged in the *C_w_* under severe hypoxia indicates that the U-shaped relationship shifted leftward only.

Another concern is that statistical power might be lower than we expected. The linear trend analysis was further applied for the data set. We addressed the concern of low statistical power by focusing on the overall slope and fit of the response in ES across the levels of hypoxia, thus reducing the number of comparisons made. This approach was particularly advantageous because changes in ES were subtle among different conditions. However, we must acknowledge that other potential factors in addition to gas exchange variables should be considered to explain the significant linear trend for the ES. For example, higher HR during exercise under hypoxia compared to normoxia, which can cause greater blood flow (BF) to exercising muscles, has been suggested to be a contributor for maintaining similar V̇O_2_ at each speed (Engelen et al., 1996).

We measured muscle deoxygenation profiles using NIRS to assess muscle O_2_ extraction indirectly. It has been suggested that HHb as an indicator of muscle O_2_ extraction (DeLorey et al., 2003; Grassi et al., 2003) could reflect the balance between muscle O_2_ utilization (V̇O_2_m) and O_2_ delivery (*Q̇*O_2_, the product of arterial O_2_ content and muscle BF). Under severe hypoxia, HHb gradually increased with increasing gait speed, showing significantly higher values above 1.250 m s^−1^ than normoxia and moderate hypoxia. We speculate that an increased leg muscle BF could compensate for a reduction in the arterial O_2_ content (CaO_2_) under moderate hypoxia, causing similar results in HHb between normoxia and moderate hypoxia. Conversely, under severe hypoxia, greater reduction in CaO_2_ could not be compensated by enhanced muscle BF, resulting in greater HHb increase compared with normoxia and moderate hypoxia.

It is well known that reduction of alveolar partial pressure of oxygen limits pulmonary O_2_ diffusion capability at high-altitude, which induces a decrease in SpO_2_ (Calbet and Lundby, 2009; Schoene, 2001). In the face of impaired pulmonary O_2_ diffusion, the rate of peripheral O_2_ delivery may play an important role in circulating arterial O_2_. It is thus possible that these differences in peripheral circulation derived by NIRS signals may affect an individual's ES in order to maintain similar V̇O_2_ at each speed. In the present study, slower ES was related to higher HHb during faster gait speeds. As shown in Fig. 3, HHb increased during faster gait speeds, in particular under severe hypoxia; therefore, the fact that muscle O_2_ extraction compensated from reduced *QO*_2_ might be partly accounted for by an individual ES.

### Methodological considerations

There are several limitations to interpret our results. First, we set 11% FiO_2_ as severe hypoxia conditions. We could not completely rule out the effect of this lower oxygen concentration on energy cost. Indeed, V̇CO_2_ was significantly higher during faster gait speeds under severe hypoxia. These results indicated that a subset of subjects were walking with a potential increase in anaerobic glycolysis to energy turnover; however, a previous study reported that V̇O_2_ manifested a delayed quasi-steady state even more than lactate threshold (Poole et al., 1988), which was a higher exercise intensity compared to our study. Thus, we believe that our main conclusion may not be strongly affected. In addition, we recruited only healthy young active subjects and performed the experiment only at level gradient. From the viewpoint of clinical implication, e.g. mountain climbing in middle-aged- and aged-populations, such information has not been available at this stage. Future studies should be warranted with various populations as well as with a larger field study.

In conclusion, moderate hypoxia at ∼15% O_2_ did not affect *C_w_* and ES during level walking in healthy young males. On the other hand, severe hypoxia at 11% O_2_ slowed the ES without changing the greater *C_w_* at faster gait speeds compared with normoxia and moderate hypoxia. From observing HHb dynamic profile under severe hypoxia, HHb responses may indicate greater O_2_ extraction rather than enhanced hypoxic-induced *QO*_2_. Thus, a significantly slower ES might be associated with hypoxic-induced higher V̇CO_2_ and greater O_2_ extraction only at severe hypoxia.

## MATERIALS AND METHODS

### Participants

Subjects were twelve fit and healthy male athletes (sprinters, middle-distance runners, soccer and baseball players), who engaged in strenuous daily training (2 h per day, 5-6 days per week). Their mean age, height, and body mass were 24±8 years, 1.74±0.06 m, and 70±10 kg, respectively (values are mean±s.d.). Researchers explained all procedures, possible risks, and benefits of participation, and obtained written informed consent from each participant. They were asked to refrain from intense physical activity on the 2 days before and from drinking any alcohol and caffeinated beverages the day before testing. This study conformed to the Declaration of Helsinki, and the Mount Fuji Research Institute ethical committee approved all study procedures (No: ECMFRI-03-2014).

### Exercise protocols

All experiments were carried out on a motor-driven treadmill, 2.21 m long and 0.88 m wide (T7000, Johnson Health Tech. Co., Ltd, Taichung Hsein, Taiwan). Under all experimental conditions, participants walked on the same treadmill, and they were free to choose their step frequency at each speed. All participants wore underwear, shirts, socks, shorts and lightweight training shoes (Abe et al., 2004). They were allowed to familiarize themselves with treadmill walking while wearing a gas collection mask during at least three preliminary practices on the same treadmill at several gait speeds and gradients (Abe et al., 2004). Inspired oxygen concentrations were set at normobaric normoxia (21%, room air), moderate hypoxia (FiO_2_; 15%, equivalent to a simulated altitude of 2700 m, at which there is increased risk of acute mountain sickness), and severe hypoxia (FiO_2_; 11%, equivalent to a simulated altitude of 5000 m, that of the highest permanent human residences on earth). Each oxygen concentration was supplied by 200-L Douglas bag with hypoxic gas generator system (see below ‘Measurements’) and performed on different days in random order, and a single blind method was used. Participants began by sitting in a chair for 10 min, and then standing for 5 min on the treadmill while baseline values were measured. They then began to walk on the treadmill. Six gait speeds were set incrementally at 0.667, 0.861, 1.056, 1.250, 1.444, and 1.639 m s^−1^. In keeping with our recent work (Abe et al., 2015), each gait speed was maintained for 4 min.

### Measurements

V̇_E_ and gas-exchange variables were measured by an online computerized breath-by-breath method (AE-310S, Minato Medical Science, Osaka, Japan). Inspired and expired gas volumes were measured using a hot wire respiratory flow system. Flow signals were electrically integrated for the duration of each breath to calculate minute ventilation. The expired fractions of O_2_ and CO_2_ were analyzed using a zirconium solid electrolyte oxygen analyzer and an infrared carbon dioxide analyzer, respectively. The standard known gases (O_2_ 15.23%, CO_2_ 4.999%, and N_2_ balance) and room air were used for the calibration of the gas analyzer. Each gas was supplied via a 200-L Douglas bag with a hypoxic gas generator system (Everest summit II, Will Co. Ltd., Tokyo, Japan). Throughout the study, participants' HR was recorded with a wireless HR monitor (POLAR RC800X, POLAR electro, Tokyo, Japan).

Local tissue oxygenation profiles of the vastus lateralis muscle were measured using NIRS (BOM-L1TRW, Omega Wave, Tokyo, Japan), as previously described (Horiuchi et al., 2015b, 2014b). This instrument uses three laser diodes (780, 810, and 830 nm), and calculates relative tissue levels of oxygenated hemoglobin (HbO_2_) and HHb according to the modified Beer-Lambert law (Kashima, 2003). NIRS optodes were placed on the lower third of the vastus lateralis muscle (10∼12 cm above the knee joint) (Koga et al., 2007). The probe holder contained one light source probe, and two detectors were placed 2 cm (detector 1) and 4 cm (detector 2) away from the source. Hb concentrations received by detector 1 were subtracted from those received by detector 2. This procedure allowed us to minimize the influence of skin blood flow (Ando et al., 2013; Horiuchi et al., 2014a), and to provide a NIRS signal traversing approximately 20 mm, because it has been reported that NIRS signals can reach a half of the depth of the distance between the probe and detector (Patterson et al., 1989).

The thigh muscle, with attached optodes and covering, was wrapped with an elastic bandage to minimize movement of the optodes while permitting freedom of movement for treadmill walking. Pen marks were made on the skin to indicate the margins of the holder so that optodes could be positioned in exactly the same place for each test. NIRS signals were measured at 1-s intervals throughout the experiment. SpO_2_ was monitored by pulse oximeter on the left middle finger every 1 min throughout the study (TM-2564G, A&D, Tokyo, Japan).

### Data analysis

Baseline values for all physiological responses (i.e. gas exchange variables, HR, SpO_2_, and NIRS signals) were averages of the last 2 min of standing prior to starting walking. A single sample with an average final 1-min pulmonary V̇O_2_ value (ml min^−1^) and V̇CO_2_ (ml min^−1^) at each gait speed were used to obtain the energy expenditure (EE) during walking, based on the following equation (Brouwer, 1957; Masschelein et al., 2012):
(1)
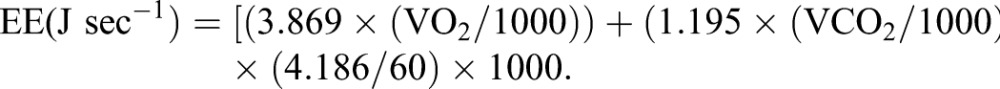
To calculate each particular *C_w_*, this equation can be transported as follows:
(2)
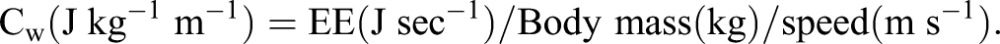
The *C_w_*-*v* relationship can be mathematically described by the following equation (Abe et al., 2015; Wall-Scheffler and Myers, 2013):
(3)
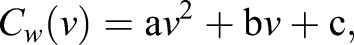
where the constants a, b, and c are determined by the least squares regressions with the actually observed *C_w_* values at each gait speed. A differential function of the original quadratic Eqn (2) of each individual can be described as follows:
(4)
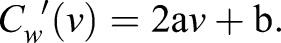
Then, the individual ES was determined at the gait speed when *C_w_*′ (*v*) equaled zero, that is, the individual ES could be observed using the following equation:
(5)
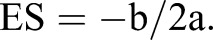
We recently reported that standing V̇O_2_ amounted approximately 50% of the absolute V̇O_2_ at the level gradient at 0.667 m s^−1^ under normoxia, indicating that a careful consideration should be necessary to calculate *C_w_* and ES by subtracting the standing metabolic rate (Abe et al., 2015). Indeed, the ES calculated including the standing metabolic rate matches preferred walking speed in many previous studies (Browning et al., 2006; Martin et al., 1992; Malatesta et al., 2003; Peyrot et al., 2012; Wall-Scheffler and Myers, 2013; Wezenberg et al., 2013). With this background, we included the standing metabolic rate to calculate *C_w_* and ES.

A single sample of the mean NIRS signals during the final 1 min at each speed was also analyzed. To compare HHb between participants, the changes in this metric were quantified as percentage changes from the baseline values. Briefly, each resting baseline value, which was represented as an arbitrary unit, was defined as 100%. Thus, changes in NIRS signals at each gait speed were represented as relative changes from baseline values. Similarly, a single sample with an average final 1 min of V̇_E_, RER, and SpO_2_ was calculated.

### Statistics

All data are presented as means±s.d. One-way repeated measures ANOVA with linear trend analysis and pairwise (Tukey) *post hoc* tests were used to evaluate the changes in cardiorespiratory variables at rest, the averaged *C_w_* during the slower and faster three stages, and the ES among different oxygen concentrations. Moreover, two-way repeated measure ANOVA was used to compare changes in the *C_w_* and the NIRS signals between different oxygen concentrations and gait speed within participants. Tukey's *post hoc* test for two-way ANOVA was employed when interactions were significant. To estimate the relationship between changes in ES and NIRS signals, a Pearson correlation coefficient was conducted. Statistical analysis was performed by commercial software packages: Sigma Stat ver 3.5 (Hulinks, IL, USA) and GraphPad Prism 7 (GraphPad Software, Inc, La, Jolla, CA, USA). A *P*-value of <0.05 was considered statistically significant.
